# 不同金属/适配体双功能复合磁性纳米材料的制备及其对外泌体的富集性能

**DOI:** 10.3724/SP.J.1123.2021.06012

**Published:** 2021-10-08

**Authors:** Weibing ZHANG, Rui LU, Lingyi ZHANG

**Affiliations:** 华东理工大学化学与分子工程学院, 上海市功能性材料化学重点实验室, 上海 200237; Shanghai Key Laboratory of Functional Materials Chemistry, School of Chemistry & Molecular Engineering, East China University of Science and Technology, Shanghai 200237, China; 华东理工大学化学与分子工程学院, 上海市功能性材料化学重点实验室, 上海 200237; Shanghai Key Laboratory of Functional Materials Chemistry, School of Chemistry & Molecular Engineering, East China University of Science and Technology, Shanghai 200237, China; 华东理工大学化学与分子工程学院, 上海市功能性材料化学重点实验室, 上海 200237; Shanghai Key Laboratory of Functional Materials Chemistry, School of Chemistry & Molecular Engineering, East China University of Science and Technology, Shanghai 200237, China

**Keywords:** 金属氧化物亲和色谱, 富集, 磁性纳米材料, 核酸适配体, 外泌体, metal oxide affinity chromatography, enrichment, magnetic nanomaterials, aptamers, exosomes

## Abstract

外泌体作为一种细胞外囊泡,其内容物可以反映亲代细胞的重要信息,而自身也具有独特的结构,能够执行特征的生物学功能。基于外泌体的表面化学和生物学特征,制备了不同类型的金属/适配体(Apt)双功能复合磁性纳米材料,并将其应用于外泌体的富集纯化。将适配体和外泌体表面目标膜蛋白的特异性结合性能与以钛、锆为代表的金属氧化物和外泌体磷脂双层膜的特异性亲和作用结合,可极大地提高分离材料对外泌体的分离选择性和富集容量。分别以Fe_3_O_4_@Zr-MOFs、Fe_3_O_4_@Zr-Ti-MOFs和Fe_3_O_4_@TiO_2_等金属有机框架(MOFs)/金属氧化物磁性纳米材料为基底,制备对应的双功能MOFs/金属氧化物-适配体复合磁性纳米材料Fe_3_O_4_@Zr-MOFs-Apt、Fe_3_O_4_@Zr-Ti-MOFs-Apt和Fe_3_O_4_@TiO_2_-Apt,并进一步对不同材料的外泌体富集性能加以评价。以超速离心法提取的模型外泌体以及尿液为样品,对修饰相同质量适配体和不同含量金属氧化物的双功能材料的富集性能加以对比。将3种双功能磁性纳米材料应用于尿液外泌体的富集,得到的外泌体裂解后经质谱鉴定,分别得到233、343和832个外泌体蛋白。这一结果也表明双功能磁性纳米材料可以充分结合核酸适配体亲和的高选择性和金属氧化物的高富集容量优势,对于复杂生物样品中外泌体的快速、高效分离纯化具有潜在的应用价值,而针对材料制备和分离纯化方法的设计也为新型外泌体富集材料的设计提供了一条可行的新思路。

## Abstract (en)

Exosomes, which are extracellular vesicles with sizes of 30-150 nm, contain proteins, lipids, RNA, etc., which can reflect important information about parental cells. They also have unique structures and can perform characteristic biological functions. Although the release of exosomes is a normal process, tumor cells release more exosomes, and the contents can induce cancer progression. Exosomes are widely distributed in body fluids at high concentrations and are easy to obtain; hence, the collection of exosomes released by tumor cells has become one of the main directions in tumor liquid biopsy. In order to ensure the reproducibility and consistency of liquid biopsy results, it is necessary to develop methods for enriching exosomes in sufficient yield and purity from complex samples. Based on the size, hydrophobic proteins, and characteristic proteins of exosomes, various methods for exosome separation and purification have been developed, such as ultracentrifugation, polymer precipitation, and immunoaffinity methods. An aptamer (Apt) is an oligonucleotide chain with a total length of 20-100 nt, which has ligand binding properties and can be used to detect different types of drugs and biomolecules at the nanomolar level. Characteristic proteins on the surface of exosomes such as CD63, CD9, and CD81 are often used as exosomes markers. At present, a variety of aptamer sequences targeting the characteristic proteins of exosomes have been reported. Zirconium and titanium cations as well as the oxides of these metals show high affinity to the phospholipid bilayer on the exosome surface and are used in the separation and purification of exosomes. Metal organic frameworks (MOFs) can provide a wealth of metal oxide affinity sites to interact with the phospholipid bilayer membrane, and their diverse organic ligands can provide numerous modification sites to bind with aptamers. In this study, different metal/aptamer dual-functional composite magnetic nanomaterials were prepared by exploiting the surface chemistry and biological characteristics of exosomes for the enrichment and purification of exosomes. Because of the specific affinity of the aptamers toward the target membrane protein on the exosome surface and the affinity of the titanium or zirconium oxide toward the phospholipid bilayer membrane of exosomes, dual-functional magnetic nanomaterials can greatly improve the enrichment capacity and separation selectivity of exosomes. Fe_3_O_4_@Zr-MOFs was used as the substrate to fabricate the dual functional MOFs/metal oxide aptamer composite magnetic nanomaterial Fe_3_O_4_@Zr-MOFs-Apt. UiO-66-NH_2_ was grown in situ on the surface of Fe_3_O_4_ by a solvothermal method to form a Zr-MOFs layer, and aptamer-CD63 was covalently bonded to the amino group of the organic ligand of the MOFs. The magnetic bimetallic metal organic framework Fe_3_O_4_@Zr-Ti-MOFs, which was fabricated via a layer-by-layer assembly approach, was used as the substrate to prepare the dual functional MOFs/metal oxide aptamer composite Fe_3_O_4_@Zr-Ti-MOFs-Apt via coordination bond formation between the metal site on the Fe_3_O_4_@Zr-Ti-MOFs and the aptamers. The third dual functional MOFs/metal oxide aptamer composite magnetic nanomaterial, Fe_3_O_4_@TiO_2_-Apt, was prepared by using Fe_3_O_4_@TiO_2_ as the substrate via coordination bond formation between the metal site on Fe_3_O_4_@TiO_2_ and the aptamers. Considering model exosomes extracted by ultracentrifugation and urine as samples, this paper compared the enrichment performance of materials modified with the same quality of aptamers and different levels of metal oxides. The dual-functional composite magnetic nanomaterials modified with different metals/aptamers were used for the enrichment of urine exosomes. The obtained exosomes were lysed and identified by mass spectrometry, and 233, 343, and 832 exosomal proteins were identified. This result also shows that dual-functional magnetic nanomaterials can fully combine the high selectivity of the nucleic acid aptamer and the high enrichment capacity of the metal oxides. The rapid, efficient separation and purification of exosomes in biological samples has excellent application potential. The material design and purification methods also provide a new idea for the development of new exosome-enrichment materials.

外泌体(exosomes)是一种几乎所有细胞都分泌的细胞外囊泡,尺寸为30~150 nm,具有磷脂双层膜结构,其表面富含多种蛋白质,其内容物包括蛋白质、核酸、脂质和代谢物^[1,2]^。外泌体是来源细胞、邻近细胞以及远处组织的通讯介体,是癌症信息的宝贵载体^[3,4,5,6,7,8]^,其在体液中广泛存在,含量丰富且易于获取^[9]^,故收集由肿瘤细胞释放的外泌体已成为肿瘤液体活检的主要方向之一^[10]^。为保证液体活检结果的可重复性和一致性,需发展从复杂样品中富集具有足够产量和纯度的外泌体方法。目前基于外泌体的尺寸、疏水性蛋白和特征蛋白发展了对应的外泌体分离纯化方法,如超速离心法、聚合物沉淀法和免疫亲和法等^[11,12,13,14]^。其中,超速离心法是外泌体研究领域公认的“金方法”,该法需进行多次高速离心,且需要昂贵的超速离心机,所得外泌体产量低、纯度高^[15]^。

适配体(aptamers, Apt)是一段总长为20~100 nt的寡核苷酸链,表现出配体结合特性,可用于在nmol水平上检测不同类型的药物和生物分子,被称为化学抗体^[16,17,18,19]^。适配体的识别特性来自于特定序列(基因型)和折叠成具有特定功能(表型)的不同形状^[20]^。除了适配体特定的结构组成,氢键、范德华力、*π*共轭效应和静电相互作用等非共价相互作用也稳定了靶分子-适配体复合物^[21]^。相较于抗体,适配体成本低,易于合成及化学修饰,可重复使用^[22,23]^。外泌体表面的特征蛋白如跨膜蛋白CD63、CD9、CD81常被作为外泌体的标记^[24]^,其中CD63是被使用最多的外泌体标记^[25]^。目前,已有文献^[26,27]^报道多种靶向外泌体特征蛋白的适配体序列。以外泌体特征蛋白作为鉴定靶标,适配体已广泛应用于外泌体检测^[25,28-32]^。Zhang等^[33]^制备了CD63适配体修饰的磁性纳米材料,可以在15 min内快速捕获且无损释放外泌体。Xie等^[34]^制备了表面带正电荷的介孔二氧化硅纳米材料,并在材料表面修饰表皮生长因子受体(EGFR)适配体,用于结合人肺癌细胞A549中高表达EGFR的外泌体,并将血液中致癌性的外泌体从小肠代谢出去。

锆、钛金属离子及其氧化物对外泌体表面磷脂双层具有高亲和力,被应用于外泌体的分离纯化。Sun等^[35]^制备了修饰钛离子和磷脂衍生物的双功能磁珠,钛离子与外泌体磷脂双层膜结合,磷脂衍生物可以插入外泌体膜内,两者协同实现尿液中外泌体的快速分离和有效富集。Zhang等^[36]^制备了Fe_3_O_4_@TiO_2_-DNA aptamer,并将其应用于快速分离尿液中的外泌体,该材料结合了TiO_2_和外泌体磷脂双层膜亲和作用与适配体和外泌体表面目标膜蛋白特异性相互作用,能够在10 min内捕获尿液中92.6%的外泌体,经质谱鉴定得到999个蛋白质。Zhang等^[37]^制备了锆基金属有机框架(MOFs)材料Fe_3_O_4_@PDA@UiO-66-NH_2_,并将其应用于外泌体和外泌体中磷酸化蛋白的连续富集,鉴定到来自255个磷酸化蛋白的707条磷酸化肽。MOFs材料是一类由金属离子或金属簇与有机配体通过配位键形成的有机-无机杂化材料。锆基和钛基MOFs材料可以提供丰富的金属氧化物亲和位点与磷脂双层作用。MOFs材料多样的有机配体可提供丰富的修饰位点,如5'端修饰有-COOH的适配体可与MOFs材料有机配体中的-NH_2_共价结合,便于制备适配体修饰的MOFs材料。

本文以3种具有丰富金属氧化物位点的磁性纳米材料Fe_3_O_4_@Zr-MOFs、Fe_3_O_4_@Zr-Ti-MOFs和Fe_3_O_4_@TiO_2_为基底,以不同质量的基底磁性纳米材料修饰相同质量的CD63适配体,制备了3类(6种)双功能磁性纳米材料。制备的双功能材料可以充分结合核酸适配体亲和的高选择性和金属氧化物的高富集容量优势,利用适配体与外泌体膜蛋白CD63和金属氧化物亲和位点与磷脂双层膜间存在特异性相互作用,实现尿液中外泌体的分离纯化。本文对比研究了上述6种双功能磁性纳米材料及相应的3种基底磁性纳米材料分离纯化尿液外泌体的差异,并与相关文献结果进行了比较。结果表明,高金属氧化物含量的3种双功能磁性纳米材料的富集性能更优异,高金属氧化物有助于提高富集容量,而适配体的引入提高了材料捕集外泌体的选择性,双功能材料更适用于复杂样品中外泌体的分离纯化。

## 1 实验部分

### 1.1 仪器、试剂与材料

X型能谱仪(APOLLO, EDAX,美国)、透射电子显微镜(JEM-1400, JEOL,日本)、Zeta View纳米颗粒跟踪仪(PMX110, Particle Metrix,德国)、超速离心机(CP80MX, Hitachi,日本)、nano LC-MS/MS质谱仪(Easy nLC1000 System, Thermo Fisher Scientific,德国)。

CD63-COOH适配体(COOH-5'-CAC CCC ACC TCG CTC CCG TGA CAC TAA TGC TA-3')、CD63-SH适配体(5'-CAC CCC ACC TCG CTC CCG TGA CAC TAA TGC TA -3'-C6 SH)购于生工生物工程(上海)股份有限公司;1-(3-二甲氨基丙基)-3-乙基碳二亚胺盐酸盐(EDC)、*N*-羟基琥珀酰亚胺(NHS)、三(2-羧乙基)膦(TCEP)、苯甲基磺酰氟(PMSF)、氯化锆(ZrCl_4_, 纯度98%)、四氯双(四氢呋喃)合钛(IV)(TiCl_4_(THF)_2_, 纯度98%)、钛酸四丁酯(TBOT)、2-氨基对苯二甲酸(H_2_BDC-NH_2_)、六水合三氯化铁(FeCl_3_·6H_2_O)、三羟甲基氨基甲烷(Tris)、无水乙酸钠(NaAc)、氨水(NH_3_·H_2_O)、乙二醇(EG)、甲酸(FA)、乙腈(ACN)、*N*,*N*-二甲基甲酰胺(DMF)、乙醇(EtOH)均购于上海阿拉丁试剂有限公司;尿素(urea)、碳酸氢铵(NH_4_HCO_3_)、硫脲(thiourea)购于国药集团化学试剂有限公司;BCA蛋白浓度测定试剂盒P0011购于上海碧云天生物技术有限公司;二硫苏糖醇(DTT, 纯度99%)、碘代乙酰胺(IAA, 纯度99%)、胰蛋白酶(trypsin)、Millipore超滤管(3000 MWCO/100000 MWCO)、0.22 μm无菌滤膜均购于美国Sigma Aldrich公司;尿液由3名健康志愿者(24~25岁女性)提供,所有采集的健康志愿者均与提供者签署了书面知情同意书,并经伦理委员会批准。

### 1.2 磁性纳米材料的制备

1.2.1 基底磁性纳米材料的制备

称取2.7 g FeCl_3_·6H_2_O和7.2 g NaAc,均匀分散于100 mL EG中,机械搅拌30 min,将溶液转移至反应釜中,于200 ℃反应8 h,清洗烘干得Fe_3_O_4_颗粒。

称取50 mg Fe_3_O_4_颗粒、75 mg ZrCl_4_和58 mg H_2_BDC-NH_2_,均匀分散于36 mL DMF中,将溶液转移至反应釜中,于120 ℃反应24 h,所得颗粒清洗烘干得Fe_3_O_4_@Zr-MOFs。

Fe_3_O_4_@Zr-Ti-MOFs的制备参考本课题组之前的工作^[38]^,称取200 mg Fe_3_O_4_颗粒,分散于50 mL 含0.015 mol/L的ZrCl_4_的DMF溶液中,于120 ℃机械搅拌30 min,所得颗粒用DMF清洗3次,再分散于50 mL含0.015 mol/L H_2_BDC-NH_2_的DMF溶液中,于120 ℃机械搅拌1 h,所得颗粒用DMF清洗3次,得到Fe_3_O_4_@(Zr-MOFs)-NH_2_;将Fe_3_O_4_@(Zr-MOFs)-NH_2_分散于50 mL含0.015 mol/L的TiCl_4_(THF)_2_的DMF溶液中,于120 ℃机械搅拌30 min,所得颗粒用DMF清洗3次,再分散于50 mL含0.015 mol/L H_2_BDC-NH_2_的DMF溶液中,于120 ℃机械搅拌1 h,所得颗粒用DMF清洗3次,得到Fe_3_O_4_@(Zr-Ti-MOFs)_1_-NH_2_。上述步骤重复10次得Fe_3_O_4_@Zr-Ti-MOFs。

称取50 mg Fe_3_O_4_颗粒,分散于90 mL EtOH、30 mL ACN和0.5 mL NH_3_·H_2_O混合溶液中,超声5 min后逐滴加入1 mL TBOT,于室温下机械搅拌1.5 h,磁分离去除上清液,颗粒重新均匀分散于40 mL EtOH和20 mL H_2_O溶液中,再将溶液转移至反应釜中,于160 ℃反应20 h,得到Fe_3_O_4_@TiO_2_。

1.2.2 双功能磁性纳米材料的制备

吸取15 μL 100 μmol/L CD63-COOH适配体于85 μL含400 mmol/L EDC、100 mmol/L NHS的10 mmol/L Tris缓冲盐溶液(TBS缓冲液,pH=5)中活化1 h。在相同的适配体缓冲液中分别加入3 mg和4 mg Fe_3_O_4_@Zr-MOFs,孵育过夜,用TBS缓冲液清洗材料,得到Fe_3_O_4_@Zr-MOFs-Apt-1和Fe_3_O_4_@Zr-MOFs-Apt-2,将其保存于TBS缓冲液中,于4 ℃备用。

吸取15 μL 100 μmol/L CD63-SH适配体和15 μL 10 mmol/L TCEP溶液,于60 μL 10 mmol/L TBS缓冲液(pH=5)中活化1 h。在相同的适配体缓冲液中分别加入1.5 mg Fe_3_O_4_@Zr-Ti-MOFs、2 mg Fe_3_O_4_@Zr-Ti-MOFs、3 mg Fe_3_O_4_@TiO_2_和4 mg Fe_3_O_4_@TiO_2_,孵育过夜,用TBS缓冲液清洗材料,得到Fe_3_O_4_@Zr-Ti-MOFs-Apt-1、Fe_3_O_4_@Zr-Ti-MOFs-Apt-2、Fe_3_O_4_@TiO_2_-Apt-1和Fe_3_O_4_@TiO_2_-Apt-2,将其保存于TBS缓冲液中,于4 ℃备用。

### 1.3 尿液样品前处理

收集志愿者的中段晨尿共计400 mL,以4 ℃ 2000 g离心20 min,用0.22 μm无菌滤膜过滤上清液,以去除细胞碎片和大量凋亡小体。最后用超滤管(100000 MWCO)浓缩滤液得到预处理后的尿液,于-80 ℃储存备用。

用超速离心法提取尿液中的外泌体,将预处理后的尿液以4 ℃ 10000 g离心30 min去除沉淀微囊泡,取上清液在150000 g下离心2 h,得到外泌体沉淀。弃上清,用TBS缓冲液清洗外泌体沉淀以除去干扰蛋白质,再以150000 g离心2 h,得到外泌体沉淀,沉淀重悬于TBS缓冲液,并作为模型外泌体于-80 ℃储存备用。

### 1.4 外泌体富集及外泌体蛋白酶解

将磁性纳米材料分散于50 μL TBS缓冲液中,加入20 μL 0.5 g/L的模型外泌体或200 μL预处理后的尿液,于4 ℃孵育15 min,磁分离去除上清液。用TBS缓冲液清洗材料并重悬材料,再加入100 μL尿素裂解液(8 mol/L尿素、2 mol/L硫脲、1 mmol/L苯甲基磺酰氟),冰浴超声30 min,磁分离取上清液得裂解后的外泌体蛋白溶液。用超滤管(3000 MWCO)将上清液置换为10 mmol/L DTT,于56 ℃水浴中还原1 h,再将溶液置换为25 mmol/L IAA,于暗处烷基化0.5 h,再将溶液置换为50 mmol/L NH_4_HCO_3_(pH=8.3)。加入适量胰蛋白酶(酶和蛋白质质量比1;40),于37 ℃酶解17 h。酶解后的肽段经除盐冻干,于-20 ℃储存备用。

对超速离心法提取的模型外泌体直接裂解,吸取100 μL尿素裂解液移至20 μL 0.5 g/L模型外泌体中,冰浴超声30 min得到模型外泌体蛋白溶液。模型外泌体蛋白酶解步骤同上。

### 1.5 分析条件

nano LC-MS/MS分析外泌体蛋白色谱条件:ReproSil-Pur C18-AQ柱(75 μm×12 cm, 3 μm);流动相A为0.1% FA水溶液,流动相B为0.1% FA乙腈溶液。梯度洗脱条件为:0~8 min, 5%B~8%B; 8~58 min, 8%B~22%B; 58~70 min, 22%B~32%B; 70~71 min, 32%B~95%B; 71~78 min, 95%B。通过搜索MaxQuant数据库鉴定外泌体蛋白。

## 2 结果与讨论

### 2.1 外泌体的表征

采用透射电子显微镜、免疫印迹和纳米颗粒追踪分析对超速离心法提取的模型外泌体进行表征。

由透射电镜图(见图1a)可以看出,外泌体形貌呈“茶托形”,体现了外泌体的双层囊膜结构,其粒径大小在30~150 nm范围内。采用纳米颗粒追踪分析表征外泌体浓度和粒径,其含量为2.1×10^12^颗粒/mL,平均直径为108.1 nm(见图1b)。在外泌体中加入适量尿素缓冲液提取外泌体蛋白,用免疫印迹检验是否存在外泌体标志性蛋白TSG101和CD9,可见两种标志性蛋白均有表达(见图1c)。另外采用BCA蛋白质定量法测定外泌体蛋白含量,从400 mL尿液中提取到176 μg外泌体蛋白。

**图1:**
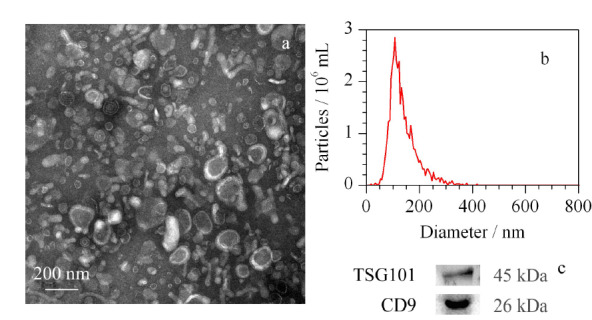
尿液外泌体的(a)透射电镜图、(b)粒径分布和(c)标志性外泌体蛋白TSG101与CD9的表达

### 2.2 双功能磁性纳米材料的制备与表征

通过X射线能谱仪表征磁性纳米材料中Zr和Ti元素的含量(见表1)。Fe_3_O_4_@Zr-MOFs中Zr元素的质量百分比为18.65%; Fe_3_O_4_@Zr-Ti-MOFs中Zr和Ti元素的质量百分比分别为25.52%和9.65%; Fe_3_O_4_@TiO_2_中Ti元素的质量百分比为23.65%。表明基底磁性纳米材料可为外泌体捕集提供丰富的金属氧化物亲和位点。

**表1:** 磁性纳米材料的基本性质

Magnetic nanomaterial	Amount of the base material/mg	w(Zr)/%	w(Ti)/%	Amount of CD63 aptamer/μg
Fe_3_O_4_@Zr-MOFs-Apt-1	3.0	18.65	0	14.67
Fe_3_O_4_@Zr-MOFs-Apt-2	4.0	18.65	0	14.67
Fe_3_O_4_@Zr-MOFs	4.0	18.65	0	0
Fe_3_O_4_@Zr-Ti-MOFs-Apt-1	1.5	25.52	9.65	14.67
Fe_3_O_4_@Zr-Ti-MOFs-Apt-2	2.0	25.52	9.65	14.67
Fe_3_O_4_@Zr-Ti-MOFs	2.0	25.52	9.65	0
Fe_3_O_4_@TiO_2_-Apt-1	3.0	0	23.65	14.67
Fe_3_O_4_@TiO_2_-Apt-2	4.0	0	23.65	14.67
Fe_3_O_4_@TiO_2_	4.0	0	23.65	0

MOFs: metal organic frameworks; Apt: aptamers.

在制备双功能磁性纳米材料时,增加基底磁性纳米材料用量会引入更多的金属氧化物亲和基团。为探究金属氧化物亲和位点和适配体在特异性富集外泌体中的作用差异,以不同质量的基底磁性纳米材料结合相同质量的CD63适配体,得到6种双功能磁性纳米材料,其基本性质列于表1。

CD63适配体是一段寡核苷酸序列,核酸在260 nm处存在紫外吸收,可通过高效液相色谱分析反应上清液中CD63适配体残余量,监测适配体与磁性颗粒结合反应进程(见图S1,详见http://www.chrom-China.com)。适配体充分反应后,上清液中CD63适配体峰消失,可以认为加入的14.67 μg CD63适配体与不同质量磁性颗粒充分结合。制备的6种双功能磁性纳米材料中CD63适配体总量保持一致。

### 2.3 模型外泌体的富集

以超速离心法提取的外泌体为模型样品评价磁性纳米材料捕集外泌体的能力,考察金属氧化物亲和位点和CD63适配体对富集效果的影响。

使用双功能磁性纳米材料富集模型外泌体时,采用在线裂解的方法获取材料富集的外泌体蛋白,即在完成富集的磁性材料中加入变性剂尿素,破坏CD63-CD63适配体复合物,洗脱捕获的外泌体。同时尿素裂解液会破坏外泌体的囊泡结构,释放其内部的蛋白质,磁分离得到的上清液即为外泌体蛋白溶液。使用基底磁性纳米材料富集模型外泌体时,同样采取在线裂解的方式,加入尿素缓冲液破坏外泌体囊泡结构,得到外泌体蛋白溶液。将得到的外泌体蛋白酶解除盐,进行nano LC-MS/MS分析。本文在相同条件下,分别采用制备的6种双功能磁性纳米材料和相应的3种基底磁性纳米材料对模型外泌体以及尿液外泌体进行捕集。

根据nano LC-MS/MS分析结果对比研究6种双功能磁性纳米材料和相应的3种基底磁性纳米材料富集模型外泌体的差异。表2列出上述9种材料富集模型外泌体所得蛋白质及模型外泌体直接裂解所得蛋白质的个数,将质谱鉴定到的蛋白质与Vesiclepedia外泌体数据库(http://www.microvesicles.org/)对比,列出归属于外泌体的蛋白质个数,蛋白质的详细信息列于表S1~表S10。

**表2:** 磁性纳米材料富集模型外泌体的质谱分析结果

Magnetic nanomaterial	Number of proteins	Number of exosomal proteins
Fe_3_O_4_@Zr-MOFs-Apt-1	135	129
Fe_3_O_4_@Zr-MOFs-Apt-2	269	243
Fe_3_O_4_@Zr-MOFs	95	82
Fe_3_O_4_@Zr-Ti-MOFs-Apt-1	158	146
Fe_3_O_4_@Zr-Ti-MOFs-Apt-2	230	212
Fe_3_O_4_@Zr-Ti-MOFs	133	123
Fe_3_O_4_@TiO_2_-Apt-1	225	204
Fe_3_O_4_@TiO_2_-Apt-2	474	431
Fe_3_O_4_@TiO_2_	212	198
Model exosomes	512	482

从鉴定所得蛋白质的归属情况可以看出上述9种材料均有特异性富集外泌体的能力,即靶向CD63的核酸适配体、MOFs材料中的金属-氧簇及金属氧化物均可应用于外泌体的分离纯化。超速离心法是公认的外泌体提取“金方法”,所得外泌体纯度高但产量低,离心后的上清液经再次离心仍能获得外泌体,耗时一般不少于6 h。而使用本文所述的磁性纳米材料,只需15 min即可完成分离纯化。

在修饰有相同质量的CD63适配体情况下,比较同类型双功能磁性纳米材料富集外泌体的差异。含有更多金属氧化物亲和位点的双功能磁性纳米材料可以提高对外泌体的富集容量,但同时也会引入杂蛋白,这归因于金属氧化物和适配体结合外泌体的原理不同。金属氧化物与外泌体的磷脂双层膜相互作用,其结合位点为外泌体的所有膜表面;而CD63适配体的结合位点仅为外泌体表面众多膜蛋白中的CD63,即使CD63在外泌体中高表达,其可结合位点也远远少于金属氧化物可结合的位点,故引入高含量的金属氧化物可以提高对外泌体的富集容量。适配体被认为是“化学抗体”,适配体-靶标复合物的解离常数大小通常为μmol~pmol^[39]^,具有高选择性。同质量的双功能磁性纳米材料与基底磁性纳米材料相比,修饰的CD63适配体可以大大提高捕获外泌体的数量,得到更多的外泌体蛋白,体现了适配体特异性富集外泌体的能力。而仅用基底磁性纳米材料富集所得外泌体蛋白数量均低于双功能磁性纳米材料,表明双功能材料可以充分结合核酸适配体亲和的高选择性和金属氧化物的高富集容量优势,从而用于外泌体的分离纯化。

比较不同类型磁性纳米材料富集外泌体的差异,3类材料结合适配体的质量均为14.67 μg,而Fe_3_O_4_@TiO_2_类相较于Fe_3_O_4_@Zr-MOFs类,质量相同但金属氧化物含量更高,富集所得外泌体蛋白个数更多,同时也引入更多的杂蛋白,再次证明了金属氧化物的高富集容量优势,后续需要优化材料中适配体的比例以调节选择性。Fe_3_O_4_@Zr-MOFs类与Fe_3_O_4_@Zr-Ti-MOFs类的富集结果未见显著差异,Fe_3_O_4_@Zr-Ti-MOFs类金属氧化物含量更高,且质量仅为Fe_3_O_4_@Zr-MOFs类1/2,故提高材料金属氧化物的含量,可以用更少的基底材料结合同等质量的适配体。

结果表明,Fe_3_O_4_@Zr-MOFs-Apt-2、Fe_3_O_4_@Zr-Ti-MOFs-Apt-2和Fe_3_O_4_@TiO_2_-Apt-2富集外泌体的性能更优异。优化金属氧化物和适配体在双功能磁性纳米材料中的含量与比例,有望进一步提高其外泌体富集能力并调节其选择性。在肿瘤液体活检中,提取到足够产量和纯度的外泌体才能获得有效的生物信息,所以具有高选择性和高富集容量的双功能材料更适用于复杂样品中外泌体的分离纯化。

### 2.4 尿液外泌体的富集

经过对磁性纳米材料富集外泌体的可行性验证,进一步将其应用于实际样品中外泌体的富集。选取尿液作为实际样品,尿液中的干扰成分包括尿调蛋白等高丰度蛋白以及大小密度与外泌体接近的脂蛋白。用nano LC-MS/MS确认9种材料富集所得尿液外泌体蛋白,表3列出质谱鉴定到的蛋白个数、归属于外泌体的蛋白个数、非外泌体的蛋白个数和游离蛋白的占比,同时与相关文献进行比较。蛋白质的详细信息列于表S11~表S19。可以看出,在复杂样品中,材料富集所得外泌体蛋白个数未见显著下降,均体现出很好的抗干扰能力。比较同质量的双功能磁性纳米材料与基底磁性纳米材料富集尿液外泌体的结果差异,根据富集组分中非外泌体的蛋白个数可以看出,在基底磁性纳米材料表面修饰适配体可以增加外泌体蛋白个数,而少有杂蛋白的引入。如Fe_3_O_4_@Zr-MOFs-Apt-2相较于Fe_3_O_4_@Zr-MOFs多鉴定出133个外泌体蛋白,只增加了12个杂蛋白,证明适配体在复杂样品中仍能折叠正确构象,保持高选择性。

**表3:** 磁性纳米材料富集尿液外泌体的质谱分析结果

Magnetic nanomaterial	Number of proteins	Number of exosomal proteins	Number of non-exosomal proteins	Percentage of non-exosomal proteins/%
Fe_3_O_4_@Zr-MOFs-Apt-1	75	69	6	8.0
Fe_3_O_4_@Zr-MOFs-Apt-2	255	233	22	8.6
Fe_3_O_4_@Zr-MOFs	110	100	10	9.1%
Fe_3_O_4_@Zr-Ti-MOFs-Apt-1	300	277	23	7.7
Fe_3_O_4_@Zr-Ti-MOFs-Apt-2	373	343	30	8.0
Fe_3_O_4_@Zr-Ti-MOFs	355	319	36	10.1
Fe_3_O_4_@TiO_2_-Apt-1	654	575	79	12.1
Fe_3_O_4_@TiO_2_-Apt-2	946	832	114	12.1
Fe_3_O_4_@TiO_2_	795	695	100	12.6
Fe_3_O_4_@UiO-66-NH_2_@PA-Ti^4+^	386	284	102	26.4
Fe_3_O_4_@TiO_2_-DNA aptamer^[36]^	999	-	-	-

-: no data.

MOFs材料的比表面积大,结合能力强,已广泛应用于蛋白质组学分析。然而,外泌体由于尺寸限制(30~150 nm)效应,使其小孔中的作用位点不能够直接与外泌体产生作用,实现捕集,也因此MOFs材料结合外泌体的能力很大程度上取决于金属氧化物的含量。Fe_3_O_4_@Zr-MOFs的金属元素含量(18.65%(Zr))低于Fe_3_O_4_@TiO_2_(23.65%(Ti)),所以富集的外泌体相对较少。而通过层层自组装策略增加MOFs材料中金属元素的含量,得到的Fe_3_O_4_@Zr-Ti-MOFs(25.52%(Zr), 9.65%(Ti))具有较高的金属氧化物含量,因此也产生了更好的富集效果。

由表3可见,当金属氧化物含量较低时,如Fe_3_O_4_@Zr-MOFs-Apt-1、Fe_3_O_4_@Zr-Ti-MOFs-Apt-1和Fe_3_O_4_@TiO_2_-Apt-1,鉴定到的蛋白质数量均低于相应的基底材料(Fe_3_O_4_@Zr-MOFs、Fe_3_O_4_@Zr-Ti-MOFs和Fe_3_O_4_@TiO_2_),这是由于基底材料的用量更大,即金属氧化物结合位点更多,故鉴定的蛋白质数量多。而当基底材料用量相当时,如Fe_3_O_4_@Zr-MOFs-Apt-2、Fe_3_O_4_@Zr-Ti-MOFs-Apt-2和Fe_3_O_4_@TiO_2_-Apt-2,双功能材料鉴定到的蛋白质数量显著高于基底材料。此外,修饰了适配体的双功能材料富集到的蛋白质中游离蛋白所占的比例均低于等量的相应基底材料,显示出适配体的高选择性。

本课题组还制备了Fe_3_O_4_@UiO-66-NH_2_@PA-Ti^4+^亲水双金属磁性纳米材料(2 mg, 14.07% (Zr), 6.92% (Ti)),用于尿液外泌体的富集,经质谱鉴定得到284个外泌体蛋白。本文制备的Fe_3_O_4_@Zr-Ti-MOFs双金属磁性纳米材料(2 mg, 25.52% (Zr), 9.65% (Ti)),用于尿液外泌体的富集,经质谱鉴定得到319个外泌体蛋白,两者富集结果差异证明提高金属氧化物含量可以提高对外泌体的富集容量,在此基础上进一步修饰适配体提高材料选择性,得到的Fe_3_O_4_@Zr-Ti-MOFs-Apt-2(2 mg, 25.52% (Zr), 9.65% (Ti), 14.67 μg CD63适配体)可以富集尿液中343个外泌体蛋白。

邓春晖课题组^[36]^制备了Fe_3_O_4_@TiO_2_-DNA aptamer(5 mg, 12.59% (Ti), 14.72 μg CD63适配体),用于富集尿液外泌体,鉴定得到999个蛋白质。本文采用Fe_3_O_4_@TiO_2_-Apt-2(4 mg, 23.65% (Ti), 14.67 μg CD63适配体)富集尿液外泌体,鉴定得到946个蛋白质,归属于外泌体的蛋白质有832个。两者富集结果未见显著差异,但在裂解外泌体步骤存在差异:邓春晖课题组依次用氨水和脱氧核糖核酸酶(DNase Ⅰ)破坏金属氧化物和适配体与外泌体间的相互作用,将外泌体洗脱后再经尿素裂解液裂解。DNase Ⅰ是一种可以消化单链寡核苷酸的核酸内切酶,CD63适配体经DNase Ⅰ水解后,无法恢复原先的序列及折叠构象,失去靶向结合CD63膜蛋白的能力。而本文使用尿素裂解液在线裂解材料捕集的外泌体,仅破坏CD63-CD63适配体复合物而不影响适配体在材料表面的固定,同时又能使外泌体磷脂双层破裂,释放外泌体蛋白。

基于金属氧化物与外泌体磷脂双层膜和磷酸化肽的磷酸根间的特异性亲和作用,该课题组还报道了将Fe_3_O_4_@PDA@UiO-66-NH_2_应用于连续富集尿液外泌体及外泌体酶解液中的磷酸化肽,鉴定得到来自255个磷酸化蛋白的707条磷酸化肽^[37]^。

将双功能材料富集的尿液外泌体与模型外泌体对比,均鉴定出模型外泌体(总离子流色谱图见图S2)中未鉴定到的外泌体蛋白。如图2所示,用Fe_3_O_4_@Zr-MOFs-Apt-2、Fe_3_O_4_@Zr-Ti-MOFs-Apt-2和Fe_3_O_4_@TiO_2_-Apt-2分别富集尿液外泌体,可以对应得到83、125和471个未在模型外泌体中鉴定到的外泌体蛋白(总离子流色谱图见图S3~图S5),其结果归因于亲和法与超速离心法提取外泌体的选择性差异。超速离心法是基于粒径差异提取外泌体。本文所述双功能磁性纳米材料结合了适配体亲和色谱法和金属氧化物亲和色谱法,靶向外泌体的特征膜蛋白和磷脂双层膜,材料表面的适配体与金属氧化物亲和基团共同作用,排除了脂蛋白等尺寸与外泌体接近的蛋白和细胞器等的干扰。此外,采用双功能材料仅需几十分钟即可完成复杂样品中外泌体捕集,而无需特殊仪器辅助。

**图2:**
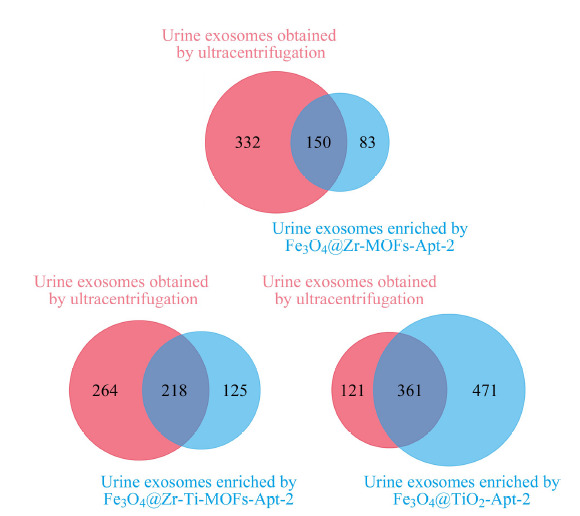
亲和法与超速离心法提取外泌体的选择性差异

## 3 结论

本文基于适配体和外泌体表面目标膜蛋白的特异性结合性能与以钛、锆为代表的金属氧化物和外泌体磷脂双层膜的特异性亲和作用,制备了不同类型的金属/适配体双功能修饰复合磁性纳米材料,并将其应用于外泌体的富集纯化。双功能材料结合适配体的高选择性和金属氧化物的高容量,为新型外泌体富集材料的设计提供了新思路。因肝脏释放的磷酸酶会将血液中的磷酸化蛋白去磷酸化,故难以识别血液中磷酸化蛋白的水平,未来可将双功能材料应用于外泌体和外泌体中磷酸化蛋白的连续富集,促进血液外泌体的磷酸化蛋白质组学研究。
